# Supplementary material for: Common Era sea-level budgets along the U.S. Atlantic coast
Source: Nat Commun. 2021 Mar 23;12:1841. doi: 10.1038/s41467-021-22079-2 (PMC7988146; doi:10.1038/s41467-021-22079-2)
Supplement: Supplementary file 1 — Supplementary Information [file 41467_2021_22079_MOESM1_ESM.pdf]

Common Era sea-level budgets along the U.S. Atlantic coast

Supplementary Figures

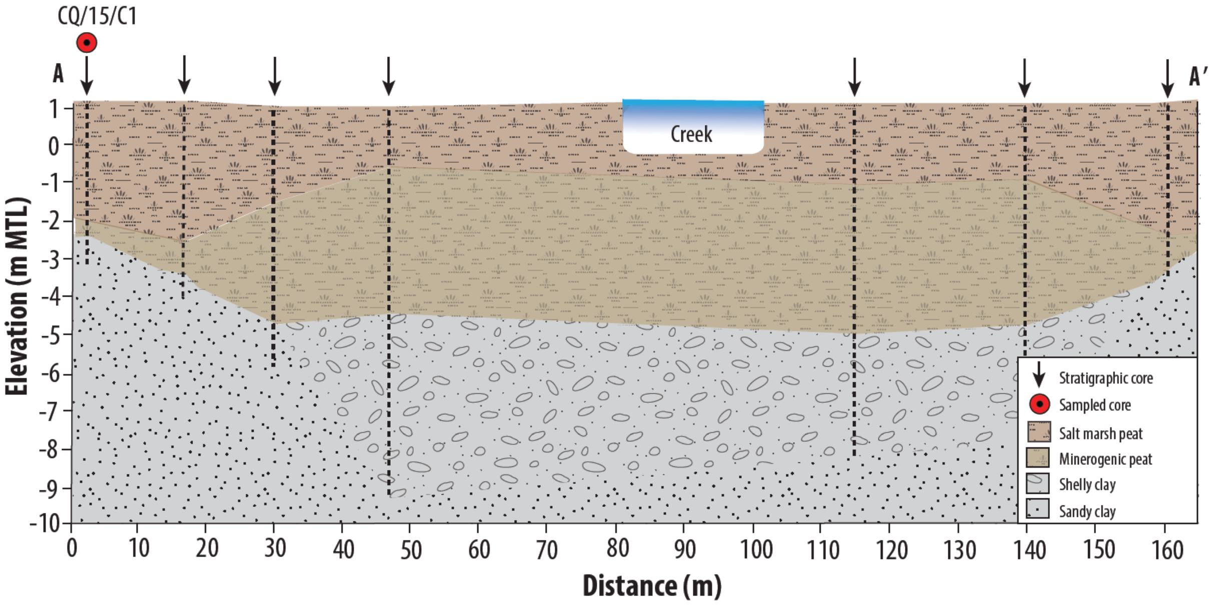

Supplementary Figure 1. Stratigraphy at Cheesequake State Park. Salt-marsh study site shows sediment cores along transect, and location of sampled core used to reconstruct relative sea level. MTL = mean tide level.

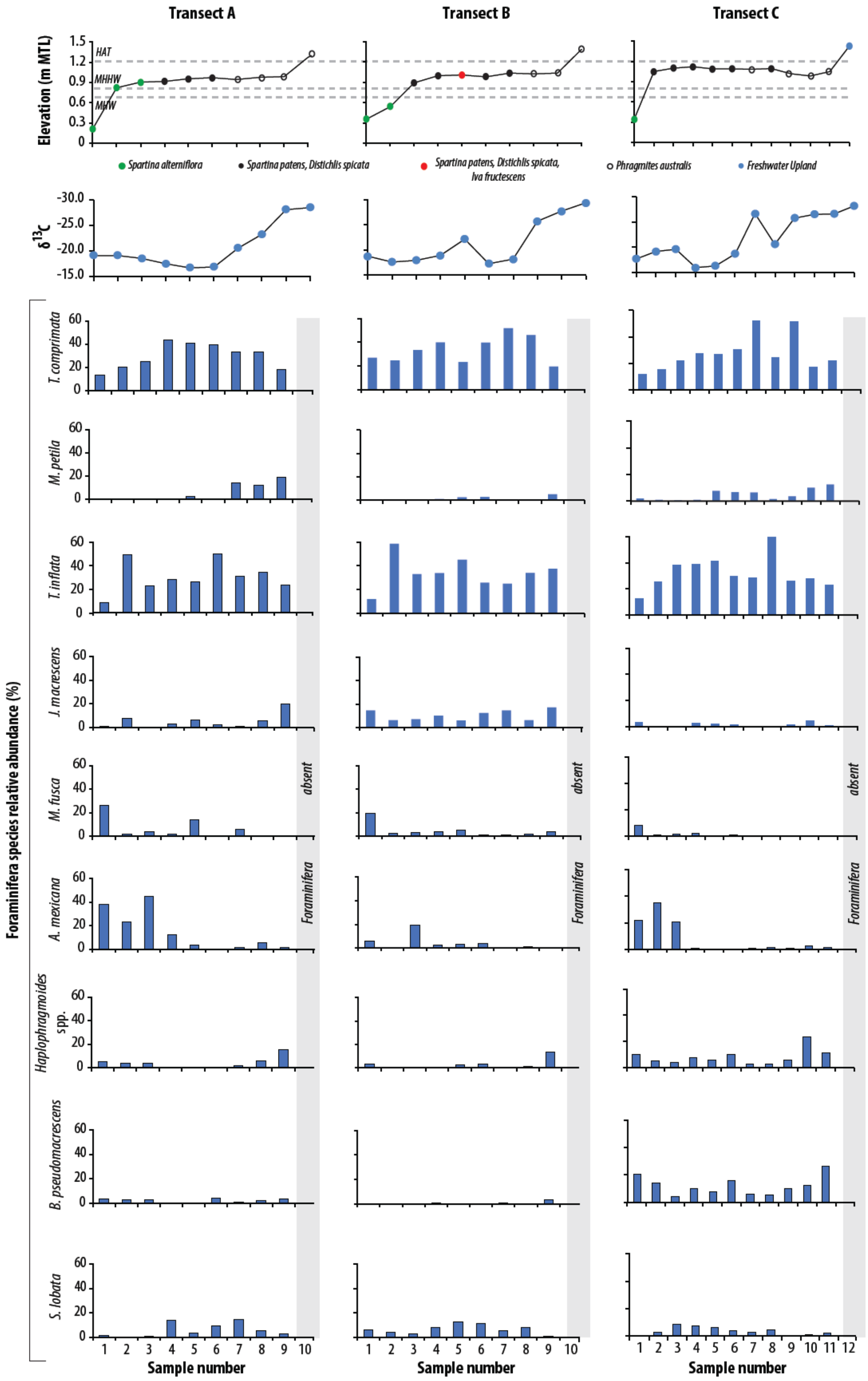

Supplementary Figure 2. Three modern transects at Cheesequake State Park. Elevation, stable carbon isotope geochemistry, and dominant dead foraminifera distributions were used in the Bayesian transfer function. MTL = mean tide level.

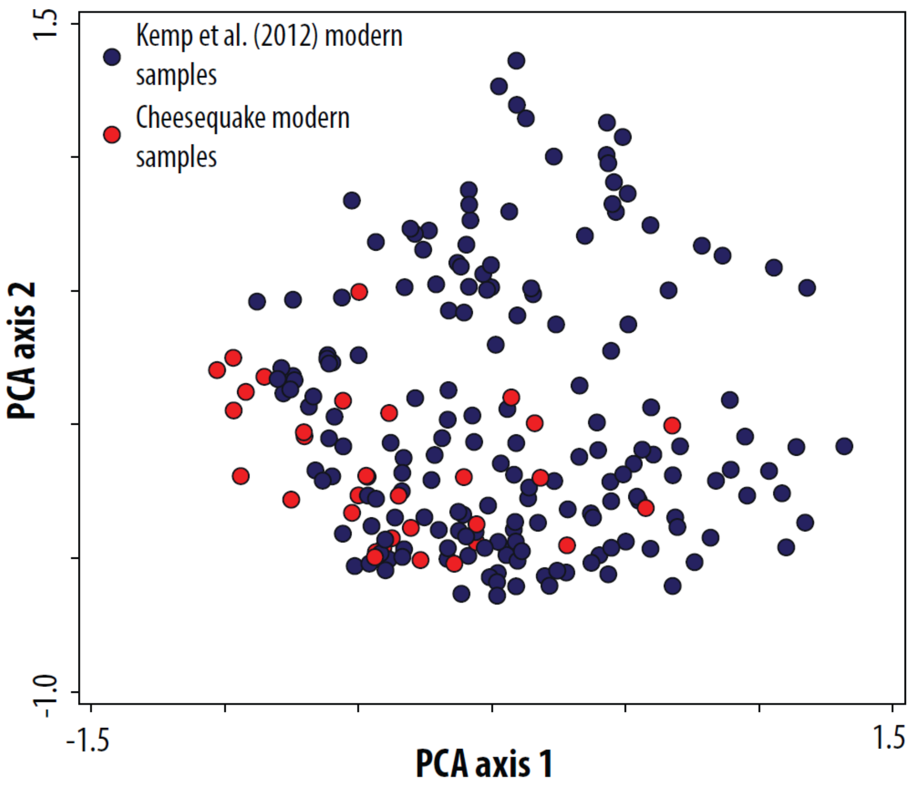

Supplementary Figure 3. Principal component analysis (PCA) of modern foraminifera assemblages. Assemblages are from southern New Jersey [1] and from Cheesequake State Park, which shows the compatibility of the two datasets because the samples fall close to one another along both first and second principal component axes.

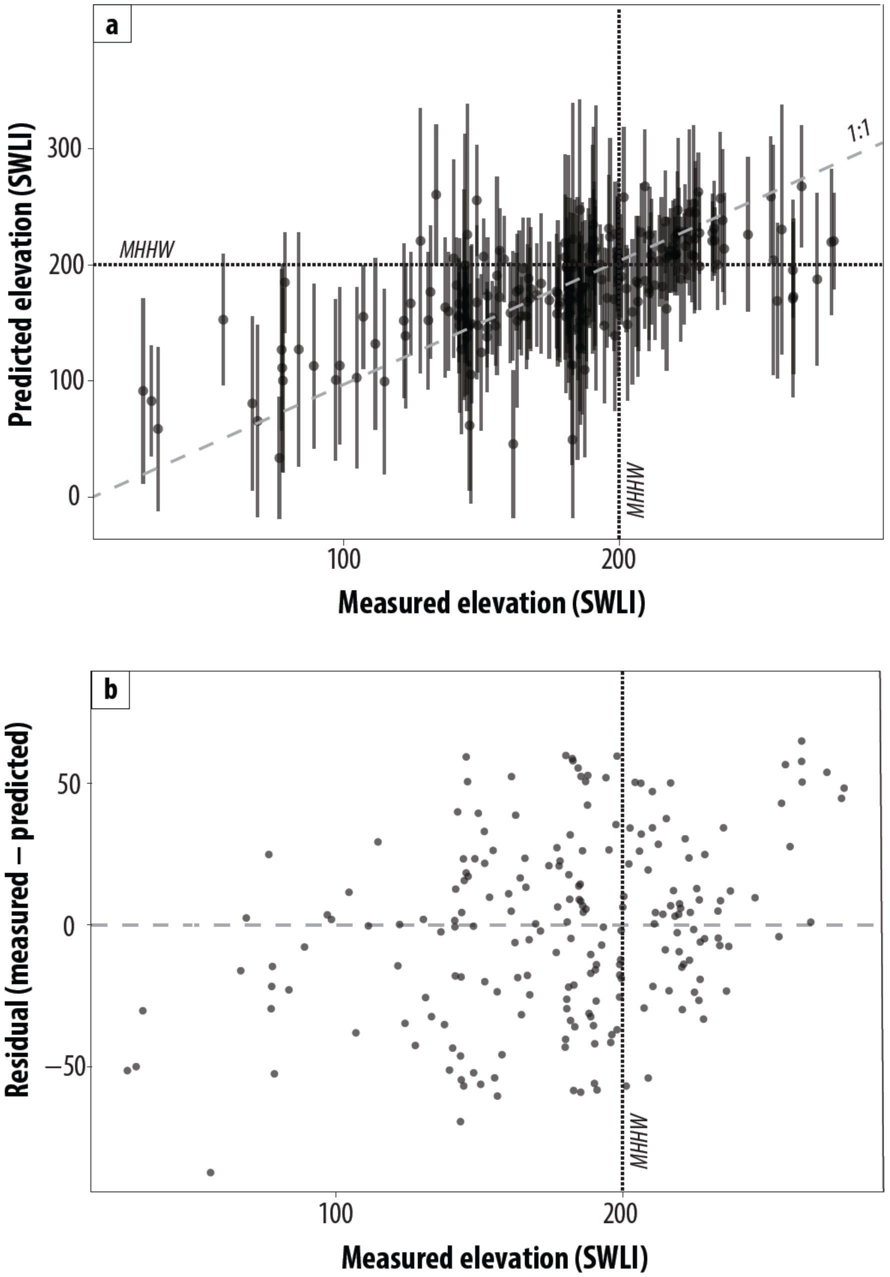

Supplementary Figure 4. Bayesian transfer function (BTF) results. (a) Predicted versus measured elevations using the BTF, which we calibrated using the combined New Jersey modern training set and evaluated its performance using 10-fold cross validation. Vertical error bars are 95% credible interval of predicted elevation. The measured elevation falls within the 95% credible intervals for 96% of the modern samples, indicating that the BTF has good predictive power. (b) Trends between residual values and measured elevations of modern samples. The absolute residuals (difference between measured and predicted elevation) average 1 standardized water level index (SWLI) unit, with a standard deviation of 32 SWLI and a maximum of 87 SWLI. The absence of a systematic trend between residual values and measured elevations of modern samples ($r^2 = 0.1$) indicates that the BTF should produce unbiased reconstructions of paleomarsh elevation (PME).

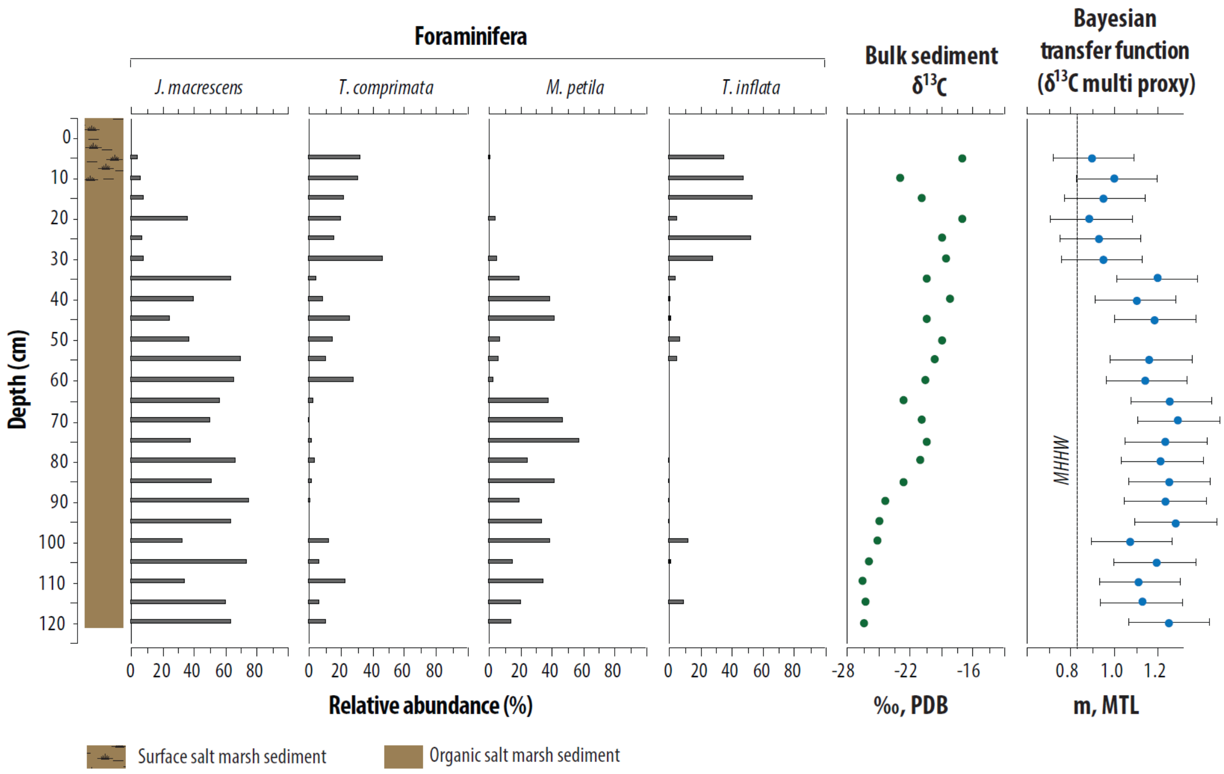

Supplementary Figure 5. Sediment core data used to reconstruct relative sea level. Dominant core foraminifera and $\delta^{13}C$ in the upper 1.2 m of the sampled sediment core (CQ/15/C1) from Cheesequake State Park. Only the four most abundant foraminifera species are shown here. The BTF was applied to the core foraminifera and $\delta^{13}C$ data to provide PME estimates with 95% credible interval for each core sample. PDB = Pee Dee Belemnite. MTL = mean tide level.

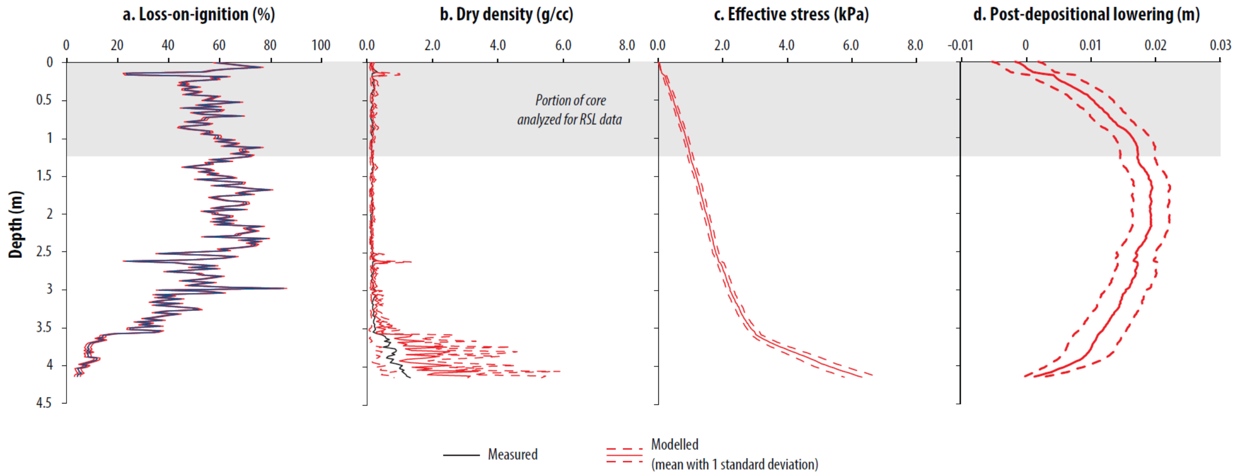

Supplementary Figure 6. Estimation of post-depositional lowering caused by sediment compaction. (a) Measured and modeled (using a geotechnical model[2–4]) loss-on-ignition (LOI) of the sampled sediment core; LOI measured after four hours at 550ºC. (b) Measured and modeled dry density of the sediment core; dry density measured after 24 hours at 105ºC. (c) Modeled effective stress through the sediment core. (d) Modeled post-depositional lowering predicted by the geotechnical model with a maximum of ~0.02 m in the middle of the core around 2 m depth.

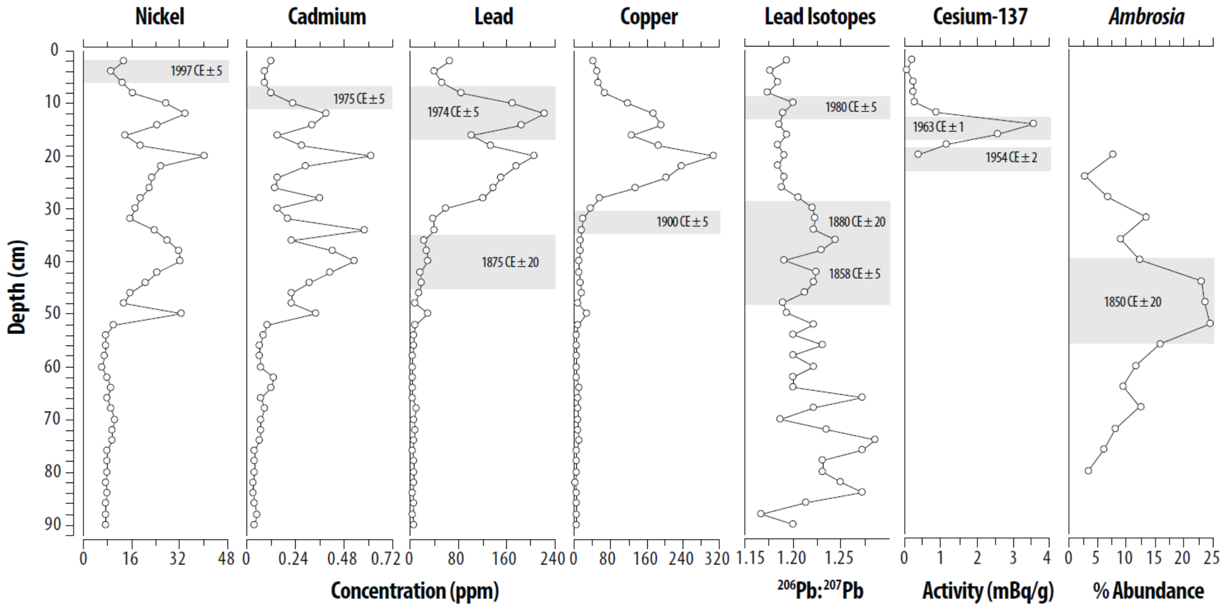

Supplementary Figure 7. Pollution and pollen chronohorizons. Changes in *Ambrosia* pollen abundances and regional-scale pollution markers, recognized in changes in down-core concentrations of lead, copper, cadmium, and nickel; the ratio of lead isotopes (^{206}Pb:^{207}Pb); and ^{137}Cs activity, which were used to provide a chronology for the upper 50 cm of the core representing the last several centuries. The markers used to build the core chronology are highlighted in grey.

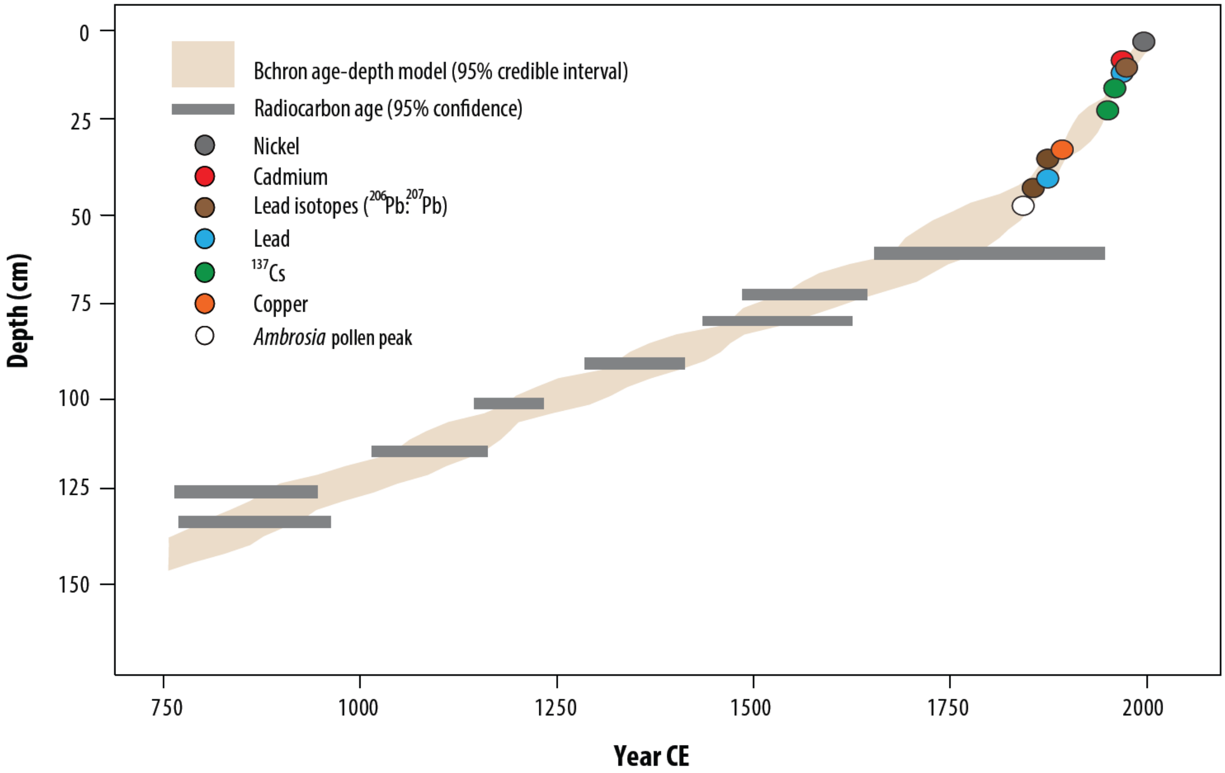

Supplementary Figure 8. Age-depth model from ~1000 CE to present. Model developed from radiocarbon dates and pollen and pollution chronohorizons, using the Bchron package in R [5,6], which uses a Bayesian framework to produce an age-depth model and estimates ages with associated uncertainties for every 1 cm thick interval in the core. The average chronological uncertainty of the relative sea-level data points is 38 years (2σ).

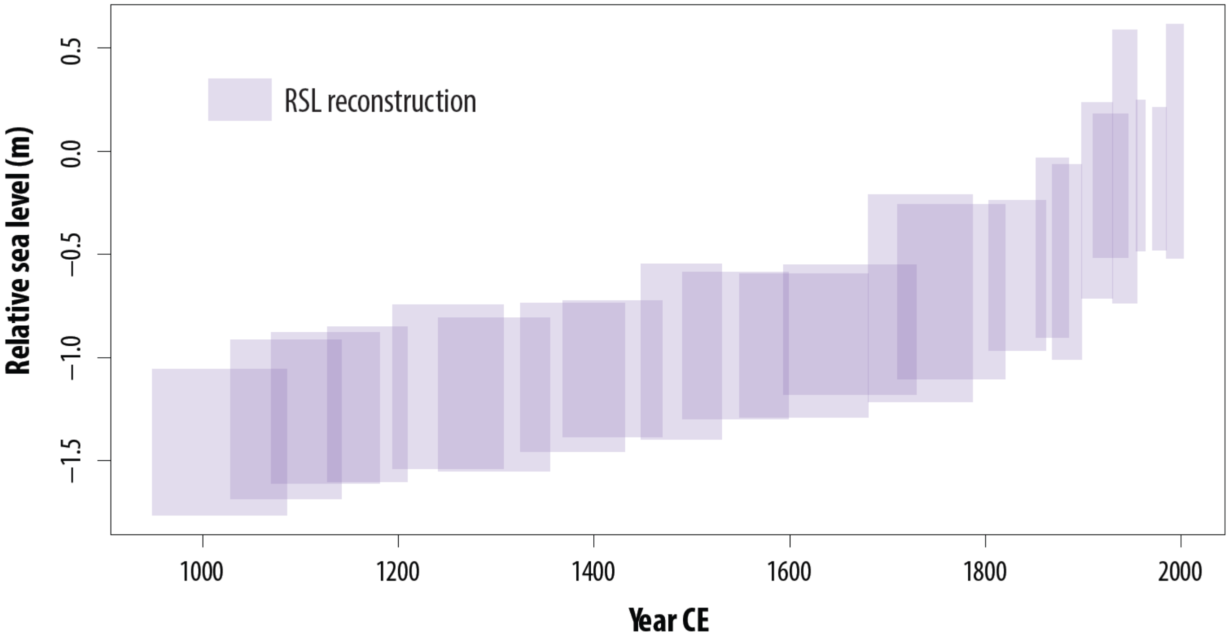

Supplementary Figure 9. Relative sea-level (RSL) reconstruction for northern New Jersey. Each box represents associated vertical relative sea level (1σ) and chronological (2σ) uncertainty for each data point.

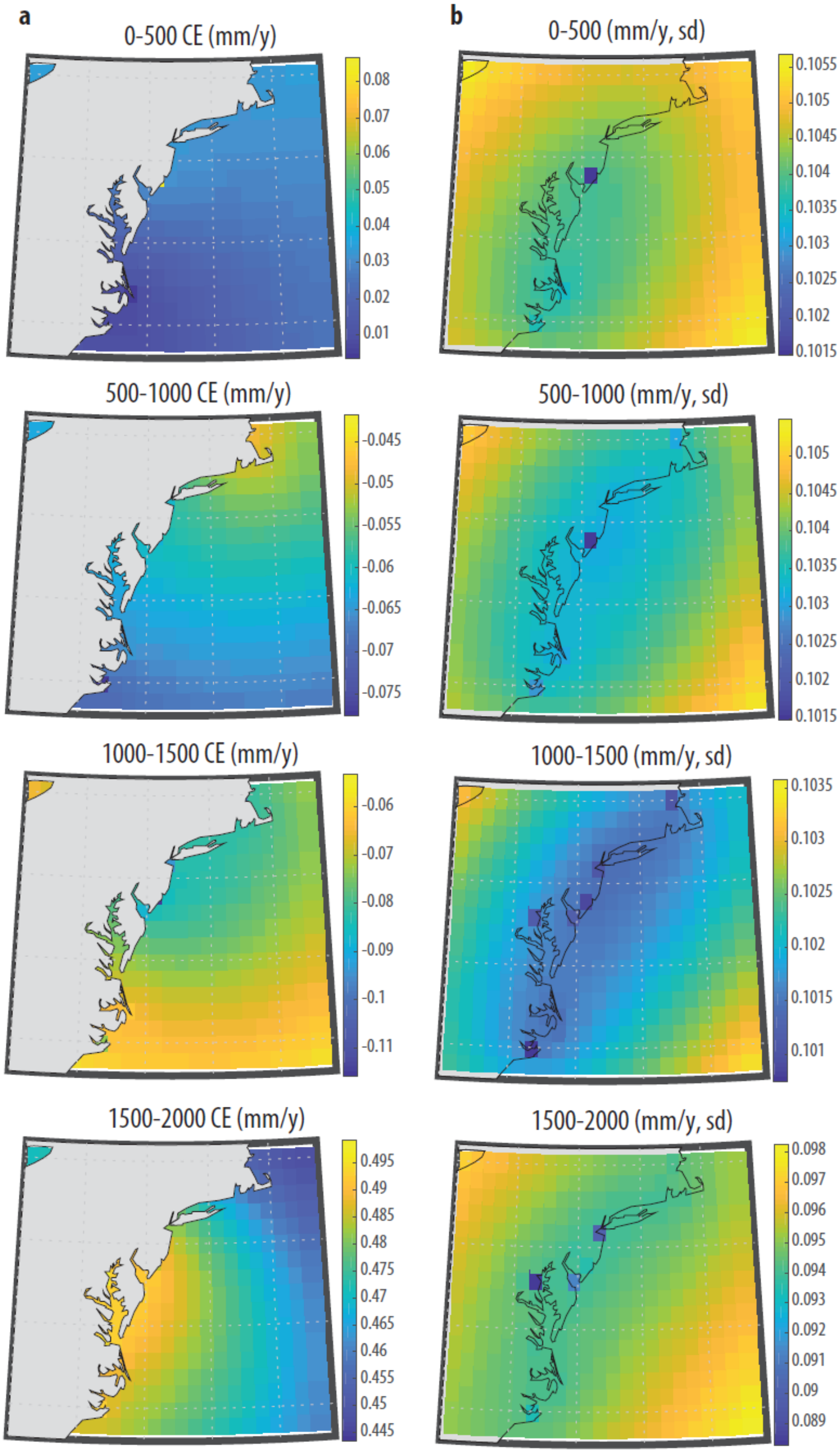

Supplementary Figure 10. Nonlinear relative-sea level change along the U.S. Atlantic coast. Spatial pattern of (a) relative sea-level change after removing the linear term $m(\mathbf{x})$ and (b) standard deviation for four time intervals. Note variable scales among panels.

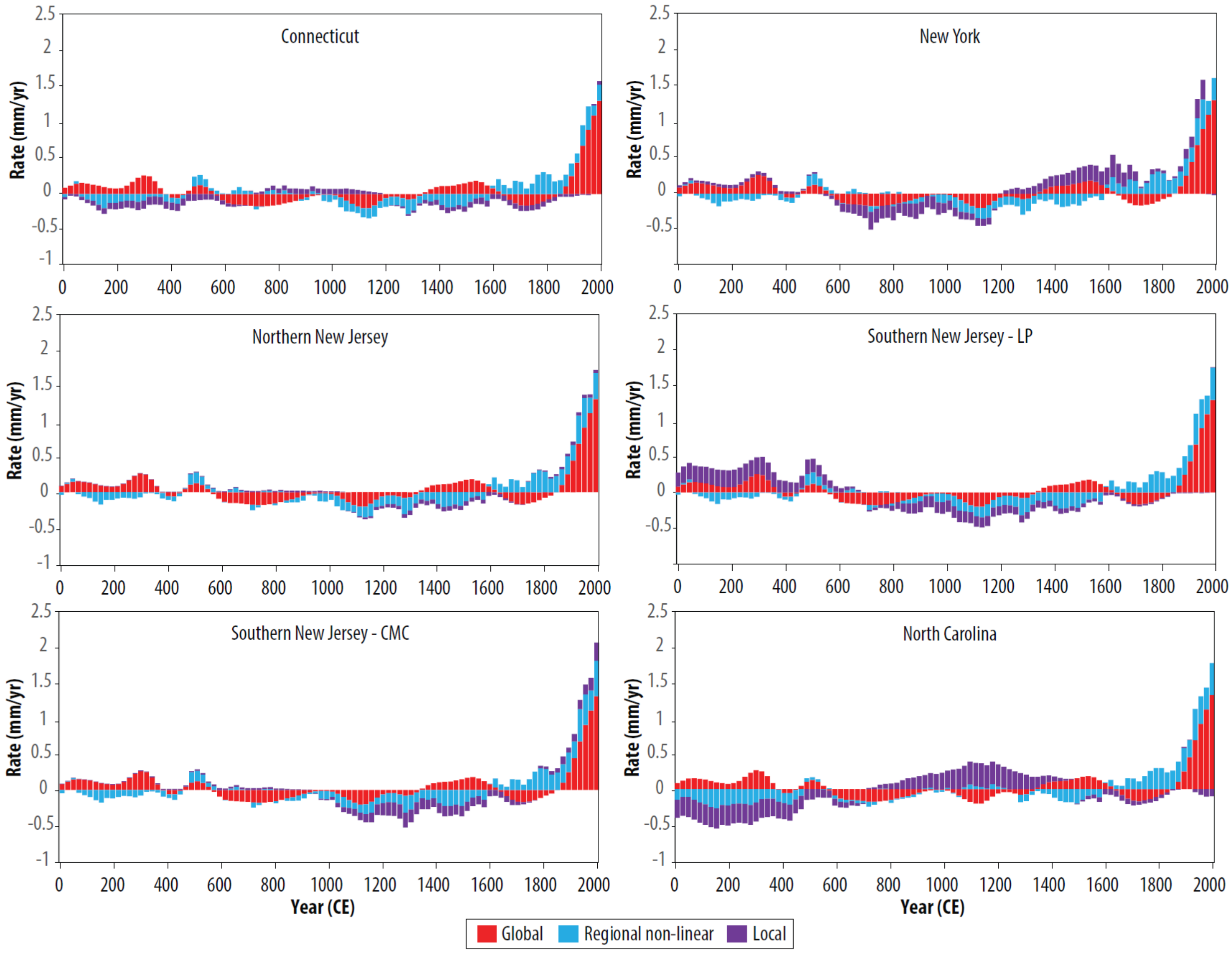

Supplementary Figure 11. Non-linear portion of Common Era sea-level budgets. Separated into global, regional, and local components for Connecticut [7], New York City [8], northern New Jersey, southern New Jersey (Leeds Point (LP) and Cape May Courthouse (CMC)) [1], and North Carolina [9].

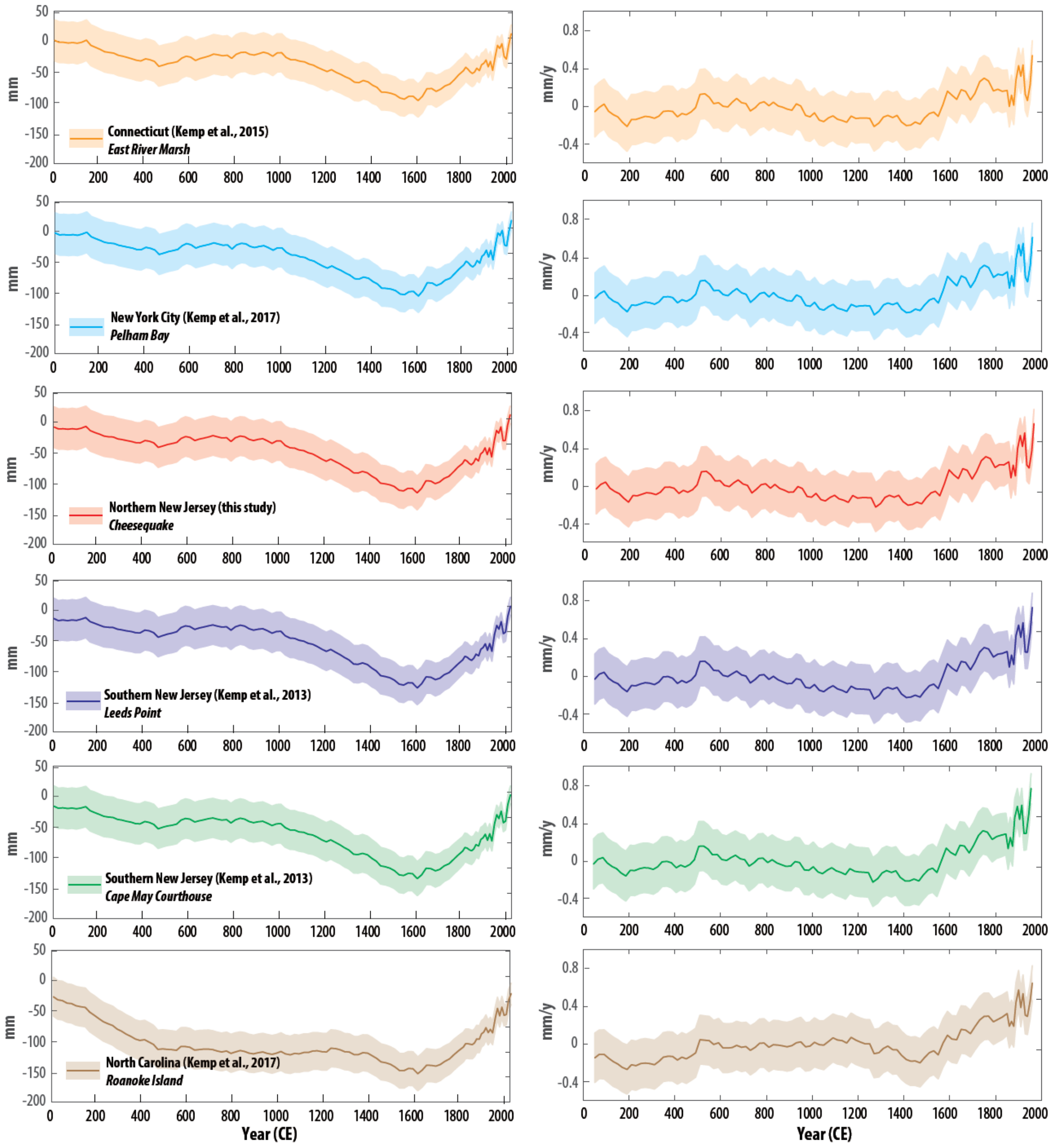

Supplementary Figure 12. Magnitudes and rates of regional non-linear component of relative sea-level decomposition. Sites are from north to south: Connecticut [7], New York City [8], northern New Jersey, southern New Jersey (Leeds Point and Cape May Courthouse) [1], and North Carolina [9]. Model predictions are the mean with 1σ uncertainty.

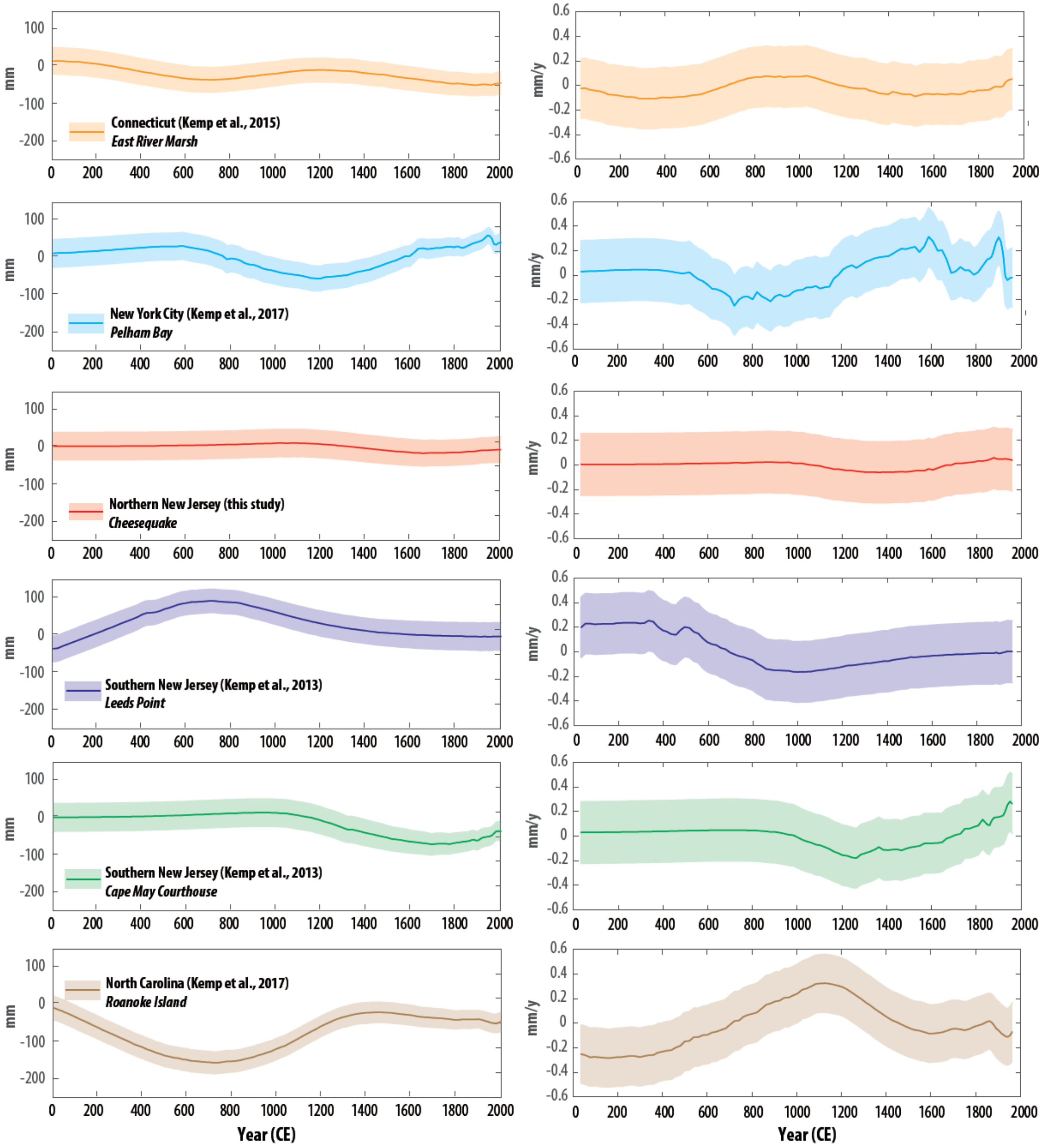

Supplementary Figure 13. Magnitudes and rates of local component of relative sea-level decomposition. Sites are from north to south: Connecticut [7], New York City [8], northern New Jersey, southern New Jersey (Leeds Point and Cape May Courthouse) [1], and North Carolina [9]. Model predictions are the mean with 1σ uncertainty.

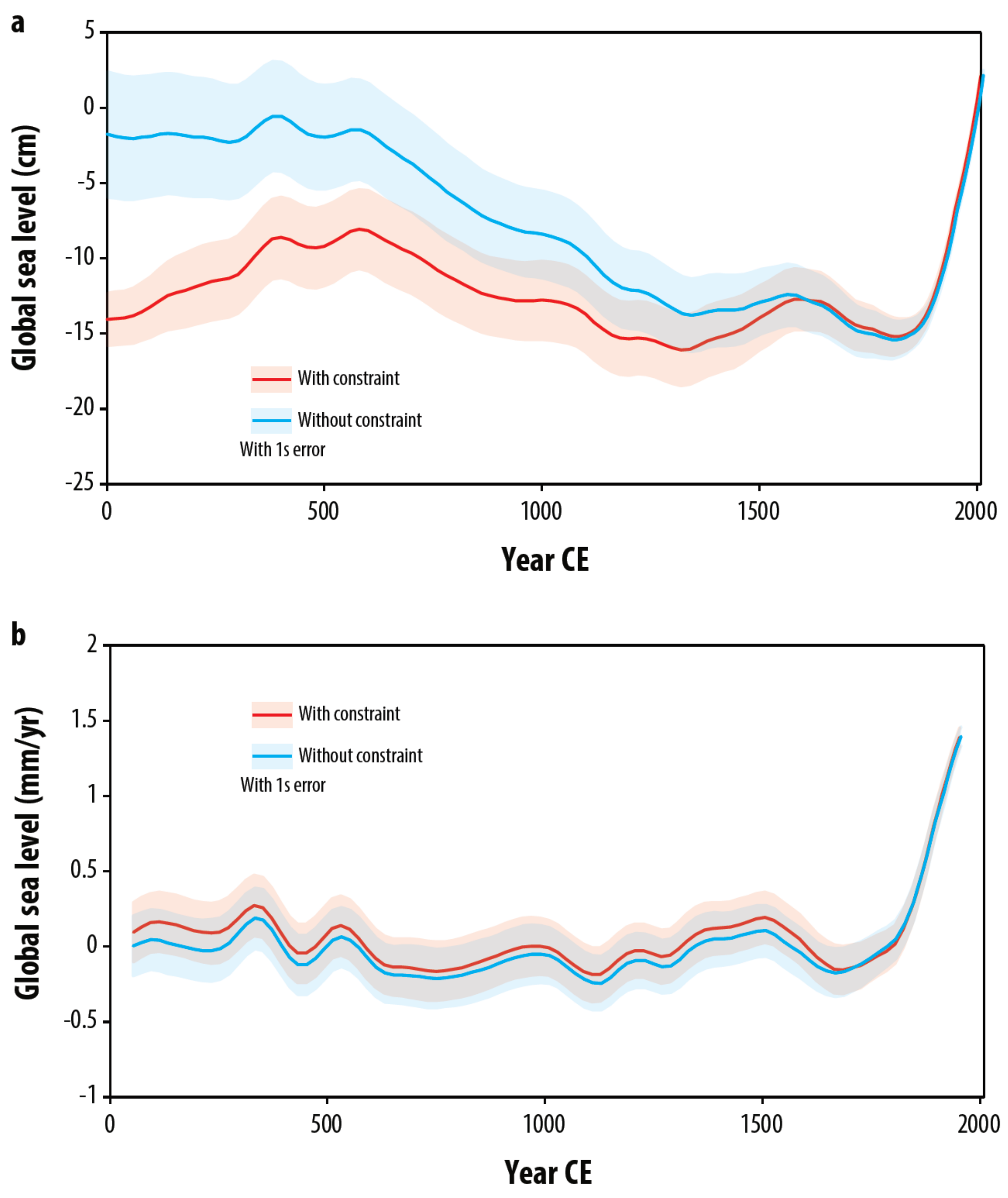

Supplementary Figure 14. Global sea-level model constraint. Reconstructed global sea level magnitudes (a) and rates (b) with and without the model constraint that mean global sea level over -100 to 100 CE is equal to mean global sea level over 1600 to 1800 CE. Model predictions are the mean with 1σ uncertainty.

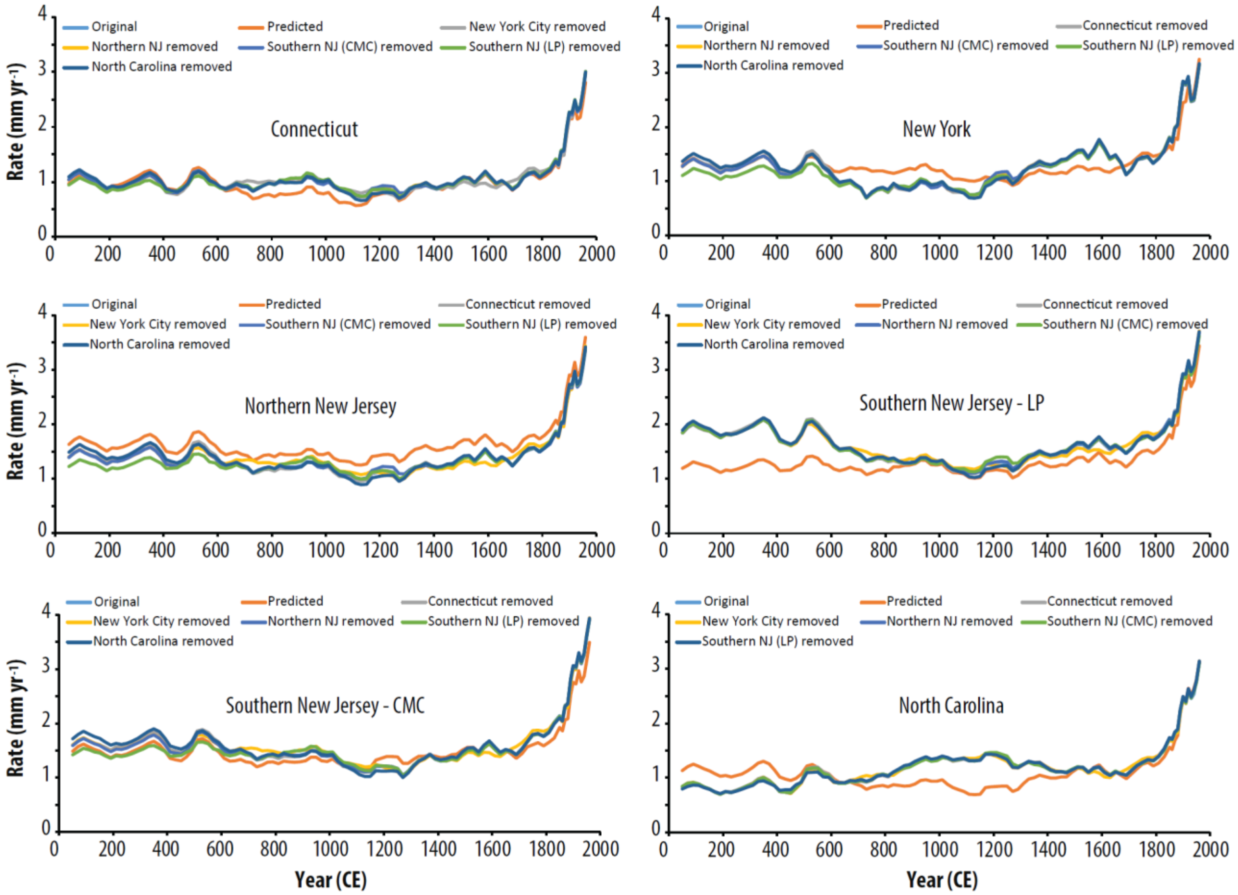

Supplementary Figure 15. Original and predicted relative sea-level rates. Rates are shown for each of the six study sites to assess the influence of removal of individual records from the database. Original rates for each site are those produced by the spatiotemporal model when that site's data are included in the database. Predicted rates are those when that site's data are removed from the database and the rates are predicted by the model using the site's lat/long coordinates. Additionally, each of the six site's data was individually removed from the database to observe the influence on the rates at the remaining five sites. LP = Leeds Point. CMC = Cape May Courthouse.

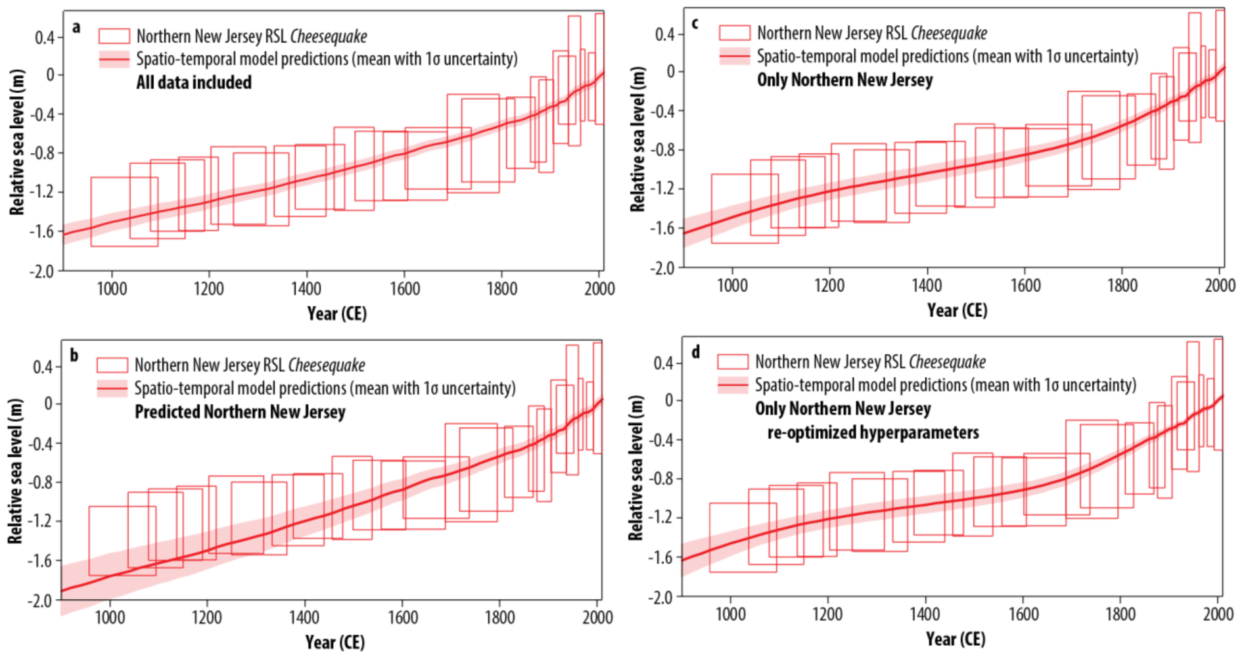

Supplementary Figure 16. A comparison of northern New Jersey relative sea-level (RSL) predictions using different model variations. (a) "All data included" is using the entire database with the model (identical to Figure 1d). (b) "Predicted Northern New Jersey" is removing the northern New Jersey data from the database, but keeping the rest of the data, and then predicting relative sea level at the northern New Jersey location (the proxy data is only shown on this figure as a reference). Here, the model is predicting northern New Jersey relative sea level and its uncertainties based on the other data in the database. The curve here is effectively the model's prior for northern New Jersey before introducing our new record, and emphasizes that the rest of the database is quite informative with respect to this prior. (c) "Only Northern New Jersey" is only using the data from northern New Jersey and removing the rest of the data in the database. In this case, there are slightly larger uncertainties in the predictions of relative sea level and a greater emphasis on shorter wavelength variability that is not supported by the corpus of sites in the region (and thus not seen in the previous case). (d) "Only Northern New Jersey," but this time with re-optimized hyperparameters, further emphasizing some centennial-scale variability that the more complete analysis smooths over. Spatiotemporal model predictions are mean with 1σ uncertainty and boxes represent the vertical RSL (1σ) and chronological (2σ) uncertainty for each data point.

Supplementary Tables

Supplementary Table 1. Reported radiocarbon ages and uncertainties from the Cheesequake State Park core with calibrated ages.

Depth (cm)	NOSAMS sample ID	Radiocarbon age (14C years)	Radiocarbon error (14C years)	Calibrated age (cal yrs. BP; 2 sigma range)
62	OS-123395	155	15	4-281
73	OS-141226	320	15	308-454
81	OS-130641	380	15	333-501
93	OS-130642	605	20	547-651
104	OS-141227	855	15	732-788
116	OS-141228	945	20	796-922
128	OS-142280	1180	20	1014-1177
136	OS-142281	1160	15	1001-1174

Radiocarbon dating was performed on identifiable plant macrofossils (stems and rhizomes) in the sediment core, which were submitted to the National Ocean Science Accelerator Mass Spectrometry (NOSAMS) facility for analysis. Each sample underwent standard acid-base-acid pretreatment at NOSAMS. Reported radiocarbon ages and uncertainties were calibrated using the IntCal13 dataset [10].

Supplementary Table 2. Optimized hyperparameters for spatiotemporal statistical model.

Term	Prior SD	Characteristic timescale (years)	Characteristic length scale (degrees)
$g_f(t)$ [fast global]	±0.1 cm	11	-
$g_s(t)$ [slow global]	±13 cm	356	-
$m(\mathbf{x})$ [linear]	±1.1 mm/yr	-	3.6
$r_f(\mathbf{x},t)$ [fast regional]	±2 cm	11	10.8
$r_s(\mathbf{x},t)$ [slow regional]	±3 cm	356	10.8
$l_f(\mathbf{x},t)$ [fast local]	±2 cm	11	0.04
$l_s(\mathbf{x},t)$ [slow local]	±3 cm	356	0.04
$w(\mathbf{x},t)$ [additional error]	±0.05 mm	-	-
$y_0(\mathbf{x})$ [datum offset]	±0.2 mm	-	-

Supplementary Table 3. Comparison of Common Era regional linear term rates using ICE5G–VM2– 90 [11] and ICE6G–VM5a [12] model predictions as prior means.

Site	GIA-predicted present-day relative sea-level rate (mm/yr)		Spatiotemporal model Common Era regional linear term rate (mm/yr)	
	ICE5G	ICE6G	ICE5G	ICE6G
Connecticut	1.0	0.9	1.032 ± 0.03	1.034 ± 0.03
New York City	1.3	1.5	1.198 ± 0.04	1.201 ± 0.04
Northern New Jersey	1.3	1.5	1.341 ± 0.08	1.345 ± 0.08
Southern New Jersey (LP)	1.4	1.7	1.596 ± 0.02	1.598 ± 0.02
Southern New Jersey (CMC)	1.2	1.6	1.553 ± 0.05	1.555 ± 0.05
North Carolina	0.7	0.9	1.155 ± 0.02	1.156 ± 0.02

Supplementary Table 4. "Leave-one-site-out" cross-validation results for the six individual sites, where the observed are the relative sea level data observations and the predicted are the model predicted relatives sea-level values at each site when its data was removed. The coverage % is the % of time that the observed relative sea-level values fall within the 95% credible intervals of the model predicted values based on the "leave-one-site-out" validation. All values are in meters. On average the model tends to over predict, especially in Southern NJ (LP) (mean error = -0.268) and in North Carolina (mean error = -0.221) which also have the largest mean absolute errors. LP = Leeds Point. CMC = Cape May Courthouse.

Site	Coverage %	Mean error (observed - predicted)	Mean absolute error
Connecticut	84	-0.149	0.149
New York	92	-0.003	0.104
Northern New Jersey	87	0.093	0.148
Southern New Jersey (LP)	96	-0.268	0.268
Southern New Jersey (CMC)	77	-0.080	0.098
North Carolina	93	-0.221	0.221

References

1. Kemp, A. C. *et al.* Sea-level change during the last 2500 years in New Jersey, USA. *Quat. Sci. Rev.* **81**, 90–104 (2013).
2. Brain, M. J., Long, A. J., Petley, D. N., Horton, B. P. & Allison, R. J. Compression behaviour of minerogenic low energy intertidal sediments. *Sediment. Geol.* **233**, 28–41 (2011).
3. Brain, M. J. *et al.* Modelling the effects of sediment compaction on salt marsh reconstructions of recent sea-level rise. *Earth Planet. Sci. Lett.* **345–348**, 180–193 (2012).
4. Brain, M. J. *et al.* Quantifying the contribution of sediment compaction to late Holocene salt-marsh sea-level reconstructions, North Carolina, USA. *Quat. Res.* **83**, 41–51 (2015).
5. Haslett, J. & Parnell, A. A simple monotone process with application to radiocarbon-dated depth chronologies. *J. R. Stat. Soc. Ser. C Appl. Stat.* **57**, 399–418 (2008).
6. Parnell, A. C., Haslett, J., Allen, J. R. M., Buck, C. E. & Huntley, B. A flexible approach to assessing synchroneity of past events using Bayesian reconstructions of sedimentation history. *Quat. Sci. Rev.* **27**, 1872–1885 (2008).
7. Kemp, A. C. *et al.* Relative sea-level change in Connecticut (USA) during the last 2200 yrs. *Earth Planet. Sci. Lett.* **428**, 217–229 (2015).
8. Kemp, A. C. *et al.* Relative sea-level trends in New York City during the past 1500 years. *The Holocene* **27**, 1169–1186 (2017b).
9. Kemp, A. C. *et al.* Extended late Holocene relative sea-level histories for North Carolina, USA. *Quat. Sci. Rev.* **160**, 13–30 (2017a).
10. Reimer, P., Bard, E. & Bayliss, A. IntCal13 and Marine13 radiocarbon age calibration curves 0-50,000 years cal BP. *Radiocarbon* **55**, 1869–1887 (2013).

11. Peltier, W. R. Global glacial isostasy and the surface of the Ice-Age Earth: The ICE-5G (VM2) model and GRACE. *Annu. Rev. Earth Planet. Sci.* **32**, 111–149 (2004).
12. Peltier, W. R., Argus, D. F. & Drummond, R. Space geodesy constrains ice age terminal deglaciation: The global ICE-6G_C (VM5a) model. *J. Geophys. Res. Solid Earth* **120**, 450–487 (2015).
